# Supplementary material for: Exploring the causal link between serum 25-hydroxyvitamin D concentrations and idiopathic sudden sensorineural hearing loss: Insights gained from a Mendelian randomization study involving two independent samples
Source: PLoS One. 2025 May 19;20(5):e0322898. doi: 10.1371/journal.pone.0322898 (PMC12087992; doi:10.1371/journal.pone.0322898)
Supplement: S1 Checklist — (DOCX) [file pone.0322898.s003.docx]

**STROBE-MR checklist of recommended items to address in reports of Mendelian randomization studies**^1^ ^2^

| **Item No.** | **Section** | **Checklist item** | **Page No.** | **Relevant text from manuscript** |
| --- | --- | --- | --- | --- |
| 1 | **TITLE and ABSTRACT** | Indicate Mendelian randomization (MR) as the study’s design in the title and/or the abstract if that is a main purpose of the study | 1-2 | **Title**: Exploring the causal link between serum 25-hydroxyvitamin D concentrations and idiopathic sudden sensorineural hearing loss: Insights gained from a Mendelian randomization study involving two independent samples  **Abstract**: Idiopathic sudden sensorineural hearing loss (ISSNHL) is defined by the rapid onset of hearing impairment without an identifiable etiology. A potential association has been suggested between reduced hearing capacity and lower serum concentrations of 25-hydroxyvitamin D (25(OH) D). However, current cross-sectional studies have only demonstrated an association, failing to establish a causal link. Therefore, a comprehensive investigation is necessary to clarify the causal relationship between them. A two-sample Mendelian randomization (MR) analysis was conducted by utilizing large-scale genome-wide association study (GWAS) summary datasets to provide information on genetically predicted serum 25(OH) D levels and the incidence of ISSNHL. Genome-wide significant (P < 1×10−8) and independent (r2 < 0.001) single-nucleotide polymorphisms (SNPs) were selected as instrumental variables (IVs). The GWAS data on serum 25-hydroxyvitamin D levels comprised 6,896,093 SNPs from 496,949 individuals of European ancestry(exposure variable). Outcome data were derived from another GWAS data, including 16,380,424 SNPs from 1,491 European ISSNHL cases and 196,592 controls. MR-Egger, inverse variance weighted (IVW), weighted median, simple mode, and weighted mode, were employed to assess causal effects. The robustness of the MR analysis results was evaluated by heterogeneity tests, horizontal pleiotropy tests, and the leave-one-out method. A total of 117 SNPs were employed as instrumental variables (P < 5 × 10−8). Our results indicated no causal association between serum 25(OH) D levels and ISSNHL within the European population (IVW; OR = 1.09, 95% CI = 0.81 to 1.48, P = 0.573). Furthermore, the statistical models did not reveal any evidence of heterogeneity or pleiotropy. |
|  | **INTRODUCTION** |  |  |  |
| 2 | **Background** | Explain the scientific background and rationale for the reported study. What is the exposure? Is a potential causal relationship between exposure and outcome plausible? Justify why MR is a helpful method to address the study question | 2 | The prevalence of ISSNHL ranges from 0.07% to 5.2%, exhibiting an age-related increase and significant variability across different countries within the adult population. Researches have indicated a possiable association between higher prevalence of vitamin D insufficiency (the exposure) and ISSNHL (the outcome).A two-sample Mendelian randomization (MR) analysis, which is inherently resistant to confounding factors, was conducted to clarify the causal relationship between them. |
| 3 | **Objectives** | State specific objectives clearly, including pre-specified causal hypotheses (if any). State that MR is a method that, under specific assumptions, intends to estimate causal effects | 3 | This study has the following specific objectives:  First, to determine the potential causal relationship between the serum levels of 25-hydroxyvitamin D and the onset of ISSNHL. We hypothesize that a genetic variant associated with levels of 25-hydroxyvitamin D will also be associated with ISSNHL, under the assumption that this genetic variant acts as an instrumental variable.  Second, to test the robustness of this causal relationship through a series of sensitivity analyses.  We clearly state that Mendelian randomization (MR) is a method that, under the assumptions of no pleiotropy, independence between genetic variants and confounders, and relevance of genetic variants to the exposure, intends to estimate causal effects. By using MR, we aim to provide more reliable evidence on the causal nature of the relationship between serum levels of 25-hydroxyvitamin D and ISSNHL, which can inform future research and interventions in audiology. |
|  | **METHODS** |  |  |  |
| 4 | **Study design and data sources** | Present key elements of the study design early in the article. Consider including a table listing sources of data for all phases of the study. For each data source contributing to the analysis, describe the following: |  |  |
|  | a) | Setting: Describe the study design and the underlying population, if possible. Describe the setting, locations, and relevant dates, including periods of recruitment, exposure, follow-up, and data collection, when available. | 4 | We conducted a two-sample MR study aimed at deriving a comprehensive and reliable conclusion regarding the causal relationship between serum 25-hydroxyvitamin D levels (the exposure) and ISSNHL (the outcome). |
|  | b) | Participants: Give the eligibility criteria, and the sources and methods of selection of participants. Report the sample size, and whether any power or sample size calculations were carried out prior to the main analysis | 4 | The exposure-related GWAS dataset for serum 25-hydroxyvitamin D levels, which included 496,946 samples of European descent and 6,896,093 SNPs, was retrieved from a publicly accessible repository, the IEU OpenGWAS Project (https://gwas.mrcieu.ac.uk, with the specific ID: ebi-aGCST90000618). The ISSNHL GWAS summary data, comprising 16,380,424 SNPs from 1,491 cases of ISSNHL and 196,592 control individuals, were also obtained from the IEU OpenGWAS Project database (GWAS ID: finn-b-H8_HL_IDIOP), which included participants of European ancestry. |
|  | c) | Describe measurement, quality control and selection of genetic variants | 5 | We ensured the independence of each SNP and minimized the potential impact of gene pleiotropy. SNPs that demonstrated a significant association with vitamin D levels were selected based on the following parameters: a genome-wide significance threshold of *P* < 5×10^-8^ and a linkage disequilibrium (LD) measure of *r*^2^  < 0.001 within a 10,000 kb window. Additionally, to mitigate bias arising from SNPs with weaker IVs, we calculated the F-statistics for each SNP using the formula: F-statistics = (Beta/Se)^2 . The resulting F-statistics values serve as indicators of the strength of the SNPs as instrumental variables, with a threshold exceeding 10 suggesting the absence of bias from weak IVs. |
|  | d) | For each exposure, outcome, and other relevant variables, describe methods of assessment and diagnostic criteria for diseases | 4 | Serum 25-hydroxyvitamin D levels can be assessed through blood tests.  Regarding idiopathic sudden sensorineural hearing loss (ISSNHL), diagnosis is typically according to the “Guidelines for Diagnosis and Treatment of Sudden Deafness (2015)”. |
|  | e) | Provide details of ethics committee approval and participant informed consent, if relevant |  | Not relevant |
| 5 | **Assumptions** | Explicitly state the three core IV assumptions for the main analysis (relevance, independence and exclusion restriction) as well assumptions for any additional or sensitivity analysis | 4 | Assumption 1: the selected instrumental variables exhibit a strong correlation with the exposure (P < 5×10-8). Assumption 2: there is no correlation between the IVs and the outcome; and Assumption 3: the IVs do not exert an influence on the outcome through confounding variables other than the exposure. |
| 6 | **Statistical methods: main analysis** | Describe statistical methods and statistics used |  |  |
|  | a) | Describe how quantitative variables were handled in the analyses (i.e., scale, units, model) | 6 | Five distinct methodologies were utilized, including inverse variance weighting (IVW), MR-Egger regression, weighted median estimation (WME), weighted mode, and simple mode, with IVW being designated as the primary analytical technique. |
|  | b) | Describe how genetic variants were handled in the analyses and, if applicable, how their weights were selected | 5 | In accordance with the criteria established in our previous Mendelian randomization studies for SNPs selection we ensured the independence of each SNP and minimized the potential impact of gene pleiotropy. SNPs that demonstrated a significant association with vitamin D levels were selected based on the following parameters: a genome-wide significance threshold of P < 5×10-8 and a linkage disequilibrium (LD) measure of r2 < 0.001 within a 10,000 kb window. Additionally, to mitigate bias arising from SNPs with weaker IVs, we calculated the F-statistics for each SNP using the formula: F-statistics = (Beta/Se)^2. The resulting F-statistics values serve as indicators of the strength of the SNPs as instrumental variables, with a threshold exceeding 10 suggesting the absence of bias from weak IVs. SNPs that fulfilled these criteria were considered appropriate for inclusion in the subsequent Mendelian randomization analysis. |
|  | c) | Describe the MR estimator (e.g. two-stage least squares, Wald ratio) and related statistics. Detail the included covariates and, in case of two-sample MR, whether the same covariate set was used for adjustment in the two samples | 6 | The IVW method assigns weights to the SNP ratios of filtered SNPs, operating under the premise that if the effect size of the instrumental variable is 0, as well as the corresponding outcome variable (the risk of ISSNHL), will also be 0.  We employed the MR-PRESSO global test and MR-Egger regression to evaluate the pleiotropy of IVs, and *P* < 0.05 indicating the presence of pleiotropy. |
|  | d) | Explain how missing data were addressed |  | No missing data were encountered during the analysis, and all included variables were complete. |
|  | e) | If applicable, indicate how multiple testing was addressed |  | Multiple testing was not applicable in this study as only one primary outcome was analyzed |
| 7 | **Assessment of assumptions** | Describe any methods or prior knowledge used to assess the assumptions or justify their validity | 5 | In accordance with the criteria established in our previous Mendelian randomization studies for SNPs selection [17], we ensured the independence of each SNP and minimized the potential impact of gene pleiotropy. SNPs that demonstrated a significant association with vitamin D levels were selected based on the following parameters: a genome-wide significance threshold of P < 5×10-8 and a linkage disequilibrium (LD) measure of r2 < 0.001 within a 10,000 kb window. Additionally, to mitigate bias arising from SNPs with weaker IVs, we calculated the F-statistics for each SNP using the formula: F-statistics = (Beta/Se)^2 [18]. The resulting F-statistics values serve as indicators of the strength of the SNPs as instrumental variables, with a threshold exceeding 10 suggesting the absence of bias from weak IVs [19]. SNPs that fulfilled these criteria were considered appropriate for inclusion in the subsequent Mendelian randomization analysis. |
| 8 | **Sensitivity analyses and additional analyses** | Describe any sensitivity analyses or additional analyses performed (e.g. comparison of effect estimates from different approaches, independent replication, bias analytic techniques, validation of instruments, simulations) | 6 | The sensitivity analysis of the results was conducted using the leave-one-out method. If the remaining SNP results, after the removal of any single SNP, fall on the correct side of the invalid line, it indicates that the removal of that SNP does not affect the overall results. This approach helps to verify the stability of the MR results. |
| 9 | **Software and pre-registration** |  |  |  |
|  | a) | Name statistical software and package(s), including version and settings used | 6 | All the analyses were performed by the R software (version 4.2.0, http://www.R-project.org; The R Foundation, Vienna, Austria), with the “Two Sample MR” packages (version 0.5.6). All results were expressed as OR and its 95% CI, with P < 0.05 considered as statistically significant. |
|  | b) | State whether the study protocol and details were pre-registered (as well as when and where) |  | The study protocol was not pre-registered. The study is based on publicly available GWAS data, and pre-registration was not considered prior to the commencement of the study. |
|  | **RESULTS** |  |  |  |
| 10 | **Descriptive data** |  |  |  |
|  | a) | Report the numbers of individuals at each stage of included studies and reasons for exclusion. Consider use of a flow diagram | 4,5 | The GWAS data on serum 25-hydroxyvitamin D levels comprised 6,896,093 SNPs from 496,949 individuals of European ancestry(exposure variable). Outcome data were derived from another GWAS data, including 16,380,424 SNPs from 1,491 European ISSNHL cases and 196,592 controls. |
|  | b) | Report summary statistics for phenotypic exposure(s), outcome(s), and other relevant variables (e.g. means, SDs, proportions) | 7 | Based on the criteria for instrumental SNP selection, 117 LD-independent SNPs were identified from the 25-hydroxyvitamin D GWAS. We examined potential confounding factors associated with these 117 SNPs and confirmed that none were related. |
|  | c) | If the data sources include meta-analyses of previous studies, provide the assessments of heterogeneity across these studies |  | The data sources for this study did not include any meta-analyses. |
|  | d) | For two-sample MR:  i.  Provide justification of the similarity of the genetic variant-exposure associations between the exposure and outcome samples  ii.  Provide information on the number of individuals who overlap between the exposure and outcome studies | 7,5,12 | i. Based on the criteria for instrumental SNP selection, 117 LD-independent SNPs were identified from the 25-hydroxyvitamin D GWAS. We examined potential confounding factors associated with these 117 SNPs and confirmed that none were related. Therefore, these 117 SNPs could be utilized in the GWAS of ISSNHL. The F-statistics for the SNPs in this study all exceeded 10, indicating a low likelihood of weak IV bias and reinforcing the reliability of the research findings. Detailed information can be found in S1 Appendix.  ii. Due to the lack of individual-level data for the exposure and outcome samples, we are unable to assess potential overlaps between these samples. This limitation is acknowledged and discussed in the context of our findings. |
| 11 | **Main results** |  |  |  |
|  | a) | Report the associations between genetic variant and exposure, and between genetic variant and outcome, preferably on an interpretable scale | 7 | associations between genetic variant and exposure, and between genetic variant and outcome were display on a Figure 1(a forest plot) |
|  | b) | Report MR estimates of the relationship between exposure and outcome, and the measures of uncertainty from the MR analysis, on an interpretable scale, such as odds ratio or relative risk per SD difference | 8 | MR estimates were shown on table one.  In this investigation, the IVW method yielded the primary result concerning causal effects (IVW; Odds Ratio = 1.09, 95% Confidence Interval = 0.81 to 1.48, P = 0.573). All regression analyses indicated a lack of causal association between serum 25-hydroxyvitamin D levels and ISHHL, as illustrated in the scatter plot (P > 0.05, Fig 3). |
|  | c) | If relevant, consider translating estimates of relative risk into absolute risk for a meaningful time period | 8 | In this investigation, the IVW method yielded the primary result concerning causal effects (IVW; Odds Ratio = 1.09, 95% Confidence Interval = 0.81 to 1.48, P = 0.573). |
|  | d) | Consider plots to visualize results (e.g. forest plot, scatterplot of associations between genetic variants and outcome versus between genetic variants and exposure) | 7 | Results were shown on forest plot (figure 2,figure3), scatterplot (figure 4) |
| 12 | **Assessment of assumptions** |  |  |  |
|  | a) | Report the assessment of the validity of the assumptions | 8 | The IVW method yielded the primary result concerning causal effects (IVW; Odds Ratio = 1.09, 95% Confidence Interval = 0.81 to 1.48, P = 0.573). All regression analyses indicated a lack of causal association between serum 25-hydroxyvitamin D levels and ISHHL, as illustrated in the scatter plot (P > 0.05, Fig 3). |
|  | b) | Report any additional statistics (e.g., assessments of heterogeneity across genetic variants, such as *I^2^*, Q statistic or E-value) | 8 | The results of the heterogeneity analysis showed that there was no heterogeneity in the IVW analysis, which was evaluated by Cohran’s Q test (P > 0.05, Table 2). |
| 13 | **Sensitivity analyses and additional analyses** |  |  |  |
|  | a) | Report any sensitivity analyses to assess the robustness of the main results to violations of the assumptions | 9 | The MR-Egger regression indicated that IVs have no pleiotropy of IVs (intercept = 0.002233724 , se = 0.00670676, P = 0.739709 (> 0.05 )).There was no outliers exist, and the funnel plot was roughly symmetrical (Fig 4A). |
|  | b) | Report results from other sensitivity analyses or additional analyses | 8-9 | The results of the heterogeneity analysis showed that there was no heterogeneity in the IVW analysis, which was evaluated by Cohran’s Q test (P > 0.05, Table 2). |
|  | c) | Report any assessment of direction of causal relationship (e.g., bidirectional MR) |  | The bidirectional MR found that SSNHL had no causal effect on serum vitamin D level. |
|  | d) | When relevant, report and compare with estimates from non-MR analyses | 10 | In a study conducted by Zandi et al. [11], a cohort comprising 50 patients diagnosed with SSNHL and 50 healthy individuals without hearing impairment, serving as a control group, was analyzed. The findings indicated a heightened prevalence of vitamin D insufficiency among individuals with SSNHL, thereby suggesting a potential link between serum vitamin D levels and the onset of SSNHL. Additionally, Szeto et al. [23] conducted a cross-sectional analysis involving 1,123 participants aged 70 years and older, which revealed that low vitamin D status was correlated with low-frequency hearing loss (LFHL) and speech-frequency hearing loss (SFHL) in the elderly population. Furthermore, a survey by Chen et al. [24] investigated the relationship between serum concentrations of 25-hydroxyvitamins D2 and D3 and hearing loss in U.S. adults, uncovering a positive correlation between serum 25(OH)D2 levels and both LFHL and SFHL within the studied group. An L-shaped relationship was also identified between serum 25(OH)D3 and LFHL and SFHL, indicating that elevated serum 25(OH)D3 levels were associated with a reduced risk of high-frequency hearing loss (HFHL) in Chen’s study. |
|  | e) | Consider additional plots to visualize results (e.g., leave-one-out analyses) | 9 | The leave-one-out analysis was performed by systematically excluding individual SNPs and then assessing the cumulative effects of the remaining SNPs. The findings indicated that the results of the remaining SNPs consistently fell to the right of the invalid line following the exclusion of any single SNP. This observation substantiates that no individual SNP exerted a disproportionate influence on the MR results (Fig 4B), thereby affirming the robustness of the MR findings. |
|  | **DISCUSSION** |  |  |  |
| 14 | **Key results** | Summarize key results with reference to study objectives | 10 | Our findings suggest that there is no causal relationship between serum vitamin D levels and ISSNHL. This outcome stands in contrast to the findings of current observational studies. |
| 15 | **Limitations** | Discuss limitations of the study, taking into account the validity of the IV assumptions, other sources of potential bias, and imprecision. Discuss both direction and magnitude of any potential bias and any efforts to address them | 11 | Firstly, the study's participant pool was predominantly composed of individuals of European descent, indicating the need for additional data collection and analysis to ascertain whether the results can be extrapolated to other ethnic groups. Secondly, hearing loss was operationally defined as a pure tone average exceeding 25 dB in either ear at low frequencies, specifically across 500, 1,000, and 2,000 Hz, as well as at higher frequencies of 4,000, 6,000, and 8,000 Hz. It is important to note that the frequency ranges utilized in this study were not exhaustively analyzed. Furthermore, the research underscores the significant role of genetic factors in the onset of ISSNHL and vitamin D deficiency, suggesting a complex interplay of origins for ISSNHL. Additionally, environmental and social factors, which are non-genetic in nature, also play a substantial role in these conditions. |
| 16 | **Interpretation** |  |  |  |
|  | a) | Meaning: Give a cautious overall interpretation of results in the context of their limitations and in comparison with other studies | 9,10,11 | While our research yielded noteworthy findings, it is crucial to recognize certain limitations. Firstly, the study's participant pool was predominantly composed of individuals of European descent, indicating the need for additional data collection and analysis to ascertain whether the results can be extrapolated to other ethnic groups. Secondly, hearing loss was operationally defined as a pure tone average exceeding 25 dB in either ear at low frequencies, specifically across 500, 1,000, and 2,000 Hz, as well as at higher frequencies of 4,000, 6,000, and 8,000 Hz. It is important to note that the frequency ranges utilized in this study were not exhaustively analyzed. Furthermore, the research underscores the significant role of genetic factors in the onset of ISSNHL and vitamin D deficiency, suggesting a complex interplay of origins for ISSNHL. Additionally, environmental and social factors, which are non-genetic in nature, also play a substantial role in these conditions; however, these elements were not comprehensively addressed in this study. Moreover, Due to the lack of individual-level data for the exposure and outcome samples, we are unable to assess potential overlaps between these samples which may leads to bias of our finding. |
|  | b) | Mechanism: Discuss underlying biological mechanisms that could drive a potential causal relationship between the investigated exposure and the outcome, and whether the gene-environment equivalence assumption is reasonable. Use causal language carefully, clarifying that IV estimates may provide causal effects only under certain assumptions | 12 | While we did not find evidence for a causal relationship between vitamin D and sudden hearing loss, it remains plausible that vitamin D could influence auditory function through mechanisms such as immune modulation or inflammatory response, which may not be captured in our analysis. |
|  | c) | Clinical relevance: Discuss whether the results have clinical or public policy relevance, and to what extent they inform effect sizes of possible interventions | 10 | This result has significant clinical implications, as it suggests that the supplementation of vitamin D may not confer protective effects against sudden hearing loss. Clinicians should exercise caution when recommending vitamin D supplementation for this purpose, as it may lead to unnecessary healthcare expenditures without demonstrable benefit. |
| 17 | **Generalizability** | Discuss the generalizability of the study results (a) to other populations, (b) across other exposure periods/timings, and (c) across other levels of exposure | 12 | Our MR-based investigation did not reveal any substantial evidence of a significant causal relationship between genetically determined serum 25-hydroxyvitamin D levels and the risk of ISSNHL among individuals of European ancestry. |
|  | **OTHER INFORMATION** |  |  |  |
| 18 | **Funding** | Describe sources of funding and the role of funders in the present study and, if applicable, sources of funding for the databases and original study or studies on which the present study is based | 13 | This study was supported by Basic and Applied Basic Research Foundation of Guangdong Province (No. 2022A1515220217) |
| 19 | **Data and data sharing** | Provide the data used to perform all analyses or report where and how the data can be accessed, and reference these sources in the article. Provide the statistical code needed to reproduce the results in the article, or report whether the code is publicly accessible and if so, where | 4-5,12 | The exposure-related GWAS dataset for serum 25-hydroxyvitamin D levels, which included 496,946 samples of European descent and 6,896,093 SNPs, was retrieved from a publicly accessible repository, the IEU OpenGWAS Project (https://gwas.mrcieu.ac.uk, with the specific ID: ebi-a-GCST90000618). The ISSNHL GWAS summary data, comprising 16,380,424 SNPs from 1,491 cases of ISSNHL and 196,592 control individuals, were also obtained from the IEU OpenGWAS Project database (GWAS ID: finn-b-H8_HL_IDIOP), which included participants of European ancestry.  The code used for the analyses is available at [GitHub repository  https://github.com/AndrewsLabUCSF/MR-tutorial] |
| 20 | **Conflicts of Interest** | All authors should declare all potential conflicts of interest | 13 | The authors have declared that no competing interests exist. |

This checklist is copyrighted by the Equator Network under the Creative Commons Attribution 3.0 Unported (CC BY 3.0) license.

1. Skrivankova VW, Richmond RC, Woolf BAR, Yarmolinsky J, Davies NM, Swanson SA, et al. Strengthening the Reporting of Observational Studies in Epidemiology using Mendelian Randomization (STROBE-MR) Statement. JAMA. 2021;under review.

2. Skrivankova VW, Richmond RC, Woolf BAR, Davies NM, Swanson SA, VanderWeele TJ, et al. Strengthening the Reporting of Observational Studies in Epidemiology using Mendelian Randomisation (STROBE-MR): Explanation and Elaboration. BMJ. 2021;375:n2233.
