# Supplementary material for: Exploring the causal link between serum 25-hydroxyvitamin D concentrations and idiopathic sudden sensorineural hearing loss: Insights gained from a Mendelian randomization study involving two independent samples
Source: PLoS One. 2025 May 19;20(5):e0322898. doi: 10.1371/journal.pone.0322898 (PMC12087992; doi:10.1371/journal.pone.0322898)
Supplement: Attachment — Submitted filename: Response to Reviewer .docx [file pone.0322898.s005.docx]

**Response to Editor**

Dear Pro. Chau

Thank you for your valuable feedback and constructive suggestions. We have carefully revised the manuscript to address all concerns and ensure compliance with PLOS ONE’s policies. Below, we provide detailed responses to your comments.

1. **Style and File Naming Compliance**

We confirm that the manuscript has been revised to fully comply with PLOS ONE’s style requirements. Formatting adjustments include proper headings, font consistency, figure/table labeling, and reference formatting.

2. **Role of Funders Statement**

As requested, we confirm the following statement for inclusion in the cover letter: This study was supported by Basic and Applied Basic Research Foundation of Guangdong Province (No. 2022A1515220217; 2021A1515110533). The funders had no role in study design, data collection and analysis, decision to publish, or preparation of the manuscript.

3. **Data Availability Policy Compliance**

We appreciate the reminder and confirm that we deposit our data before acceptance.

4. **Reference List Review**

We have meticulously reviewed all references to ensure accuracy and completeness. None of the cited papers are retracted. All references are current, relevant, and properly formatted according to PLOS ONE guidelines.

**Response to Reviewers**

**Reviewer #1:**

Dear Professor,

Thank you sincerely for your thorough review of our manuscript, we address each of your comments point-by-point (seeing below), please find our detailed responses and corresponding modifications highlighted in blue within the revised manuscript.

Once again, we are grateful for your insightful suggestions, which have significantly improved the quality of this research.

Sincerely,

Ying Zhao

On behalf of all authors

**1.** **The manuscript does not report OLS estimates for comparison. Comparing OLS and IV estimates based on Mendelian randomization (MR) could be potentially useful in discussing the omitted-variable bias in conventional OLS estimates.**

Thank you for your insightful comments. We appreciate your suggestion regarding the comparison of OLS and IV estimates based on Mendelian randomization (MR) to discuss potential omitted-variable bias in conventional OLS estimates.

However, we would like to clarify that the GWAS data we currently use for Mendelian randomization analysis are unsuitable for conducting OLS analysis. The primary reason is that the data was publicly available and the nature of the variables involved do not meet the necessary assumptions required for a valid OLS estimation. Specifically, the key independent variables in our study are endogenous due to potential reverse causality and unobserved confounding factors. Given these characteristics, OLS estimates would likely be biased and inconsistent, rendering them unreliable for meaningful comparison with the IV estimates derived from the Mendelian randomization approach.

Moreover, the MR method we employed is specifically designed to address these endogeneity issues by using genetic variants as instruments, which allows us to obtain more robust and unbiased estimates of the causal effects. Therefore, focusing solely on the IV estimates derived from MR is more appropriate for our research question and the data at hand.

We understand the importance of discussing omitted-variable bias, and we have thoroughly addressed this issue in our manuscript by discussing the potential limitations of our MR approach and the assumptions underlying the use of genetic instruments (line 88-96). We believe that this discussion provides valuable context for interpreting our results and understanding the potential biases that may still exist.

2. **The frequency ranges for hearing loss were not exhaustively analyzed in the manuscript. This may overlook variations in how hearing loss manifests across different frequency ranges.**

Thank you for your valuable feedback. We appreciate your suggestion regarding the analysis of hearing loss frequency ranges, which is indeed an important aspect of hearing loss research.

In response to your comment, we have added a discussion on the potential variations in hearing loss manifestations across different frequency ranges (line 330-339). We acknowledge that different frequency ranges may be associated with distinct pathological mechanisms, and future studies could benefit from a more detailed analysis of frequency-specific hearing loss. However, due to the limitations of our current dataset, we were unable to perform a stratified analysis based on frequency ranges. Our study focused on the overall causal relationship between serum 25-hydroxyvitamin D concentrations and ISSNHL, which typically involves multiple frequency ranges. We believe that our findings provide valuable insights into the overall causal relationship between vitamin D in ISSNHL, even without frequency-specific analysis.

We have revised the manuscript to include a discussion on this limitation and have highlighted the need for future research to explore the potential frequency-specific effects of vitamin D on hearing loss (line 373-382).

**3. My view is that key confounding variables, such as osteoporosis or parathyroid hormone fluctuations, could affect vitamin D levels and auditory health.**

We sincerely appreciate this insightful comment. While we initially employed PhenoScanner V2 during our MR analysis phase (when the tool was fully operational) to exclude SNPs associated with osteoporosis, parathyroid hormone levels, and other confounders (*P* < 5×10^-8^ ; *r*^2^ < 0.001 within a 10,000 kb window), we acknowledge the current technical limitations of this platform. To rigorously address potential pleiotropy in the revised analysis, we implemented LDtrait (LDlink-NIH) as an alternative and complementary approach to examine whether the genetic instruments (SNPs) used in our MR analysis are associated with potential confounders, including osteoporosis and parathyroid hormone levels. After a thorough check, we found that none of the SNPs were significantly associated with these confounders. Therefore, we believe that the impact of these confounders on our results is minimal (line 141-149).

Additionally, we performed sensitivity analyses using MR-Egger regression and weighted median methods to further assess the robustness of our findings. The results were consistent with our primary inverse-variance weighted (IVW) analysis, indicating that our conclusions are unlikely to be biased by unmeasured confounding.

4. **The IV estimation utilizing MR, based on genetic information, identifies the LATE (Local Average Treatment Effect) for compliers, provided that key conditions such as the monotonicity assumption hold (https://doi.org/10.1002/hec.3828). This issue should be highlighted in the revised paper.**

Thank you for this important comment. We fully agree that the IV estimation in our MR study identifies the Local Average Treatment Effect (LATE) for compliers, and this interpretation relies on key assumptions such as monotonicity. We appreciate the opportunity to clarify this point in the revised manuscript.

In our study, the IV estimates based on MR reflect the causal effect of serum 25-hydroxyvitamin D concentrations on ISSNHL among compliers—individuals whose vitamin D levels are influenced by the genetic variants used as instruments. This effect (LATE) may not generalize to the entire population, as it specifically applies to the subgroup of individuals whose exposure is affected by the genetic instruments.

The monotonicity assumption, which implies that the genetic variants used as instruments do not cause any individual to decrease their vitamin D levels if they would otherwise increase them (or vice versa), is a key condition for interpreting our results as LATE. In our study, we selected genetic variants strongly associated with serum 25-hydroxyvitamin D levels based on prior GWAS evidence, and we believe this supports the plausibility of the monotonicity assumption. However, we acknowledge that this assumption cannot be directly tested and should be considered when interpreting our findings.

In the revised manuscript, we will add a clear discussion of the LATE interpretation and the monotonicity assumption in the Methods (line 171-178) and Discussion sections (line 340-352). We will emphasize that our results apply specifically to compliers and may not represent the average causal effect in the general population.

5. **My reading is that the empirical analysis does not account for seasonal variations in serum vitamin D levels. This could impact the estimation results that are reported in the manuscript.**

Thank you for your valuable comment. We appreciate your concern regarding the potential impact of seasonal variations in serum vitamin D levels on our estimation results.

In response to your comment, we would like to highlight that our study used Mendelian randomization analysis, which relies on genetic instruments to estimate the causal effect of serum 25-hydroxyvitamin D concentrations on ISSNHL. Since genetic variants are randomly assigned at conception and remain stable throughout an individual's lifetime, they are not influenced by external environmental conditions or behavioral changes. This property makes genetic IVs particularly powerful in minimizing confounding bias, which is a major limitation in traditional observational studies. By leveraging genetic variants as IVs, MR provides a framework for estimating causal effects that are less susceptible to the distortions caused by unmeasured or residual confounding, and enables cleaner and more reliable causal inference[Lawlor DA, Harbord RM, Sterne JA, Timpson N, Davey Smith G. Mendelian randomization: using genes as instruments for making causal inferences in epidemiology. Stat Med. 2008 Apr 15;27(8):1133-63. doi: 10.1002/sim.3034. PMID: 17886233.]. So we believe our results are inherently robust to seasonal variations in vitamin D levels.

However, we acknowledge that seasonal variations in vitamin D levels could influence observational studies. To address this point, we have added a discussion in the revised manuscript to clarify the robustness of our MR results to seasonal changes and to emphasize the advantages of using genetic instruments in this context (line 310-329).

If data on the timing of sample collection (season or month) become available in future studies, it would be valuable to explore whether seasonal variations modulate the relationship between vitamin D and hearing loss. We have included this as a suggestion for future research in the revised manuscript (line 382-390).

6. **MR assumes that genetic variants influence the outcome exclusively through the exposure variable. Any violations of this assumption could compromise the empirical findings that are reported in the manuscript.**

Thank you for raising this important point regarding the core assumption of Mendelian randomization (MR) analysis. We fully acknowledge that the validity of our findings relies on the assumption that the genetic variants influence the outcome exclusively through the exposure variable . In our study, we have taken the following steps to address potential violations of this assumption:

(1) We conducted five distinct methodologies in our MR analysis, including inverse variance weighting (IVW), MR-Egger regression, weighted median estimation (WME), weighted mode, and simple mode.
(2) The IVW method presupposes the validity of all SNPs included in the MR analysis and aggregates the Wald ratios of each SNP to derive an overall weighted effect.
(3) We employed the MR-PRESSO global test and MR-Egger regression to evaluate the pleiotropy of IVs, and indicated that IVs have no pleiotropy of IVs (intercept = 0.002233724, se = 0.00670676, *P* = 0.739709 (> 0.05)).
(4) We employed the leave-one-out method to evaluate the sensitivity analysis of the results, helping to verify the stability of the MR results.
(5) We performed heterogeneity tests and showed that there was no heterogeneity in the IVW analysis, which was evaluated by Cohran’s Q test (*P* > 0.05, Table 2).
7. **The empirical analysis is restricted to individuals of European ancestry. This limits the generalizability of the findings to other ethnic groups. (See also point 8 below.)**

Thank you for raising this important question. To provide a comprehensive response, we will address this inquiry in conjunction with our reply to Point 8 in the following section.

**8. What is the external validity of the estimation results? This issue is related to the use of genetic information to estimate LATE (see also point 4 above). Are there practical policy conclusions that stem from the MR estimates?**

Thank you for raising such valuable concerns. We understand your concern about whether our conclusions can be applied to other populations. Indeed, there are differences in genetic background, lifestyle, and environmental factors among different populations, all of which may influence the relationship between vitamin D levels and sudden sensorineural hearing loss (SSNHL). However, the advantage of Mendelian randomization analysis is that it uses genetic instrumental variables to simulate a randomized controlled trial, thereby reducing the impact of confounding factors to some extent. Nevertheless, we acknowledge the importance of verifying our conclusions in other populations. Currently, there is indeed a shortage of GWAS data on vitamin D levels and ISSNHL in populations other than Europeans. This limitation restricts our ability to directly validate our findings in these populations using the same Mendelian randomization approach.

However, we believe our study still provides valuable insights for future research and clinical practice. Specifically, our findings in the European population suggest that vitamin D levels may not play a causal role in ISSNHL, which could inform future research directions and clinical guidelines in other populations. We recommend that researchers in other regions prioritize the collection and analysis of relevant GWAS data to validate our findings. In the meantime, clinicians should continue to consider individual patient characteristics and existing evidence when managing patients with ISSNHL.We look forward to future research that can further explore this relationship in diverse populations.

**Reviewer #2:**

Dear Pro. Herkenhoff

Thank you for your thorough evaluation of our manuscript and for your encouraging feedback. We sincerely appreciate your recognition of the study’s originality, methodological rigor, and clinical relevance. Your positive remarks on the robustness of the Mendelian randomization design and sensitivity analyses further validate our efforts to ensure the reliability of the findings.

We are particularly grateful for your emphasis on the implications of our conclusions for clinical practice, especially regarding the cautious interpretation of vitamin D supplementation in the context of ISSNHL. Your acknowledgment of the study’s contribution to addressing this knowledge gap is deeply motivating for our team.

Thank you once again for your time, expertise, and constructive engagement with our work. We look forward to contributing to this important dialogue through publication.

Sincerely,

Ying Zhao

On behalf of all authors

**Reviewer #3:**

**The paper is interesting and well written. The authors investigated the potential association between reduced level of vitamin D and reduced hearing capacity through a two-sample Mendelian randomization analysis. I suggest to discuss the role of vitamin D and microbioma in immune responses including chronic inflammatory immune mediated diseases (see and add as referecnes papers by Murdaca et al concerning the role of vitamin D and microbioma in immune responses including chronic inflammatory immune mediated diseases).**

Dear Pro. Murdaca

We sincerely appreciate your insightful suggestion to explore the interplay between vitamin D, the microbiome, and immune-mediated mechanisms in the context of ISSNHL. As recommended, we have expanded the Discussion section to address some points and incorporated key references to strengthen our mechanistic interpretation (line 353-368). We propose a hypothetical “vitamin D–microbiome–immune axis” to explain ISSNHL risk, which is vitamin D sufficiency could maintain gut barrier integrity, thereby reducing bacterial translocation and subsequent endotoxin-induced cochlear microvascular inflammation - a hypothesized mechanism in ISSNHL pathogenesis.

Thank you again for this insightful recommendation, which has significantly enriched the translational implications of our research.

Sincerely,

Ying Zhao

On behalf of all authors
