# Supplementary material for: Exploring the causal link between serum 25-hydroxyvitamin D concentrations and idiopathic sudden sensorineural hearing loss: Insights gained from a Mendelian randomization study involving two independent samples
Source: PLoS One. 2025 May 19;20(5):e0322898. doi: 10.1371/journal.pone.0322898 (PMC12087992; doi:10.1371/journal.pone.0322898)
Supplement: Attachment — Submitted filename: Response to Reviewers.docx [file pone.0322898.s006.docx]

**Response to Reviewers**

**Reviewer #1: I am happy with the revisions made in the paper and with the effort you put in replying to my comments and to the other two reviewers.**

Dear Professor:

We sincerely thank you for your constructive feedback throughout the revision process. Your insightful suggestions have significantly strengthened the methodological rigor and clarity of our work. We deeply appreciate your recognition of the revisions and remain grateful for their valuable contributions to strengthening this work.

**Reviewer #2: The manuscript presents a well-conducted Mendelian randomization study to investigate the causal relationship between serum 25(OH)D levels and idiopathic sudden sensorineural hearing loss (ISSNHL). The authors have used appropriate methods, addressed potential confounding factors, and performed thorough sensitivity analyses. The responses to the reviewer's comments are well-reasoned and demonstrate a good understanding of the methodology.**

**However, I would suggest the authors to explicitly state the limitations regarding the usage of public data. Since the data is publicly available and the nature of the variables involved do not meet the necessary assumptions required for a valid OLS estimation.**

**Overall, this is a valuable contribution to the field.**

Dear Professor:

We sincerely thank you for raising this critical methodological consideration. We agree that comparing OLS and IV estimates can theoretically illuminate omitted-variable bias in observational studies. However, in our Mendelian randomization framework using publicly available GWAS summary data, such a direct comparison faces inherent limitations.

We have therefore restructured the Discussion_Limitations section**(Line 389-401)** to explicitly state: while conventional OLS regression could theoretically quantify confounding bias, the use of publicly available GWAS summary data fundamentally limits our ability to implement OLS analysis. These datasets lack individual-level measurements of key confounders and lifestyle factors required for valid OLS estimation. More critically, the endogenous relationship between serum 25(OH)D and ISSNHL—whereby hearing loss could influence vitamin D status through reduced outdoor activity (reverse causality), renders OLS estimates biologically implausible in this context.

Thank you for your valuable suggestions to strengthened the methodological rigor and clarity of our work.

**Reviewer #3: The authors revised the paper accordingly to reviewers' suggestions. The revised paper may be acceptable for publication**

Dear Pro.Murdaca:

We sincerely thank you for your constructive feedback, which has significantly improved the quality of our manuscript. We deeply appreciate your recognition of the revisions and remain grateful for their valuable contributions to strengthening this work.
